# Therapeutic Effects of Synthetic Triblock Amphiphilic Short Antimicrobial Peptides on Human Lung Adenocarcinoma

**DOI:** 10.3390/pharmaceutics14050929

**Published:** 2022-04-24

**Authors:** Danjing Yang, Liang Zhu, Xiangyu Lin, Jiaming Zhu, Yusheng Qian, Wenhui Liu, Jianjun Chen, Chuncai Zhou, Jing He

**Affiliations:** 1 Department of Pathology and Pathophysiology, Shanghai Skin Disease Hospital, School of Medicine, Tongji University, Shanghai 200092, China; djyang@tongji.edu.cn (D.Y.); liangzhuphd@163.com (L.Z.); linxiangyu668@163.com (X.L.); zjm13323140016@163.com (J.Z.); 1950773@tongji.edu.cn (W.L.); 2011233@tongji.edu.cn (J.C.); 2 School of Materials Science and Engineering, Tongji University, Shanghai 201804, China; 1951656@tongji.edu.cn

**Keywords:** antimicrobial peptides, tumoricidal capacities, structure–activity relationship, membrane disruption, lung cancer

## Abstract

Because of their unique properties, antimicrobial peptides (AMPs) represent a potential reservoir of novel anticancer therapeutic agents. However, only a few AMPs can kill tumors with high efficiency, and obtaining inexpensive anticancer AMPs with strong activity is still a challenge. In our previous work, a series of original short amphiphilic triblock AMP (K_n_F_m_K_n_) analogues were developed which were demonstrated to exert excellent effects on bacterial infection, both in vitro and in vivo. Herein, the overall objectives were to assess the potent tumoricidal capacities of these analogues against human lung cancer cell line A549 and the underlying mechanism. The results of the CCK-8 assay revealed that the precise modification of the peptides’ primary sequences could modulate their tumoricidal potency. In the tumoricidal progress, positive charge and hydrophobicity were the key driving forces. Among these peptides, K_4_F_6_K_4_ displayed the most remarkable tumoricidal activity. Furthermore, the excellent anticancer capacity of K_4_F_6_K_4_ was proven by the live/dead cell staining, colony formation assay, and tumor growth observations on xenografted mice, which indicated that K_4_F_6_K_4_ might be a promising drug candidate for lung cancer, with no significant adverse effects in vitro or in vivo. In addition, the cell apoptosis assay using flow cytometry, the morphology observations using the optical microscope, confocal microscopy using CellMask™ Deep Red staining, and scanning electron microscope suggested that membrane disruption was the primary mechanism of its antitumor action. Through analyzing the structure–activity relationship, it was found that the amount of positive charge required for K_n_F_m_K_n_ to exert its optimal tumoricidal effect was more than that needed for the antimicrobial activity, while the optimal proportion of hydrophobicity was less. Our findings suggest that further analysis of the structure–activity relationship of AMPs’ primary sequence variations will be beneficial. Hopefully, this work can provide guiding principles in designing peptide-based therapeutics for lung cancer.

## 1. Introduction

Lung cancer is a major public health problem across the world [1]. It is known that the great adverse effects of and drug resistance to chemotherapy lead to poor prognosis, low survival rate, and high tumor recurrence rate [2,3,4]. Recently, significant advances in surgical techniques and new therapeutic interventions, such as targeted therapies and immune checkpoint blockades, have been found to prolong survival time and improve the quality of life of lung cancer patients [5,6,7]. Unfortunately, dubious curative effects and costly expenses hold back their application [8,9]. Therefore, it is of great importance to develop novel drugs with high efficiency, low toxicity, low drug resistance, and inexpensive cost for the treatment of lung cancer.

Antimicrobial peptides (AMPs) are a class of immune effectors produced by diverse species of animals, plants, and insects to defend themselves against bacteria, fungi, and viruses [10,11,12,13]. Owing to their emphasis on antibiotic resistance and low toxicity, AMPs have gained much interest during recent years [14,15]. While AMPs are well-known for their ability to kill microbes, there has been a rapid increase in AMPs that have been characterized to target cancers [16,17], thereby offering a new, exciting perspective. Notably, only a fraction of natural or mimetic AMPs have been confirmed to demonstrate clear anticancer activities [18,19]. On the other hand, the isolation of both natural AMPs and synthetic AMPs is time-consuming and expensive, so these approaches cannot provide patients with sufficient amounts of AMPs [20]. Currently, the main aim of this research channel is directed towards the design of AMPs with high activity, low toxicity, and low cost. Furthermore, the activity of these cancer-targeting peptides depended largely on the structure of AMPs [21,22,23]. Thus, a deeper understanding of the structure–activity relationship of AMPs is necessary for the development of new candidates.

Previously, we designed and synthesized a series of novel triblock amphiphilic short AMPs (K_n_F_m_K_n_: K_2_F_6_K_2_, K_3_F_6_K_3_, K_4_F_6_K_4_, and K_4_F_8_K_4_) which were demonstrated to exhibit evident therapeutic effects on bacterial infection, both in vitro and in vivo [24]. We extended our investigations to the antitumor ability of these analogues against the human lung cancer cell line A549 and its underlying mechanisms. In a systematic study, it was found that these peptides were successfully developed with dual functions. K_4_F_6_K_4_ was suggested as a promising drug candidate for lung cancer, since it displayed remarkable tumoricidal activity both in vitro and in vivo, without significant adverse effects. Then, it was revealed that the anticancer effects were mediated by the membrane disruption mechanism, in which the main driving forces were electrostatic attraction and hydrophobic interaction. Finally, compared with the antimicrobial activity, the connections between the primary sequence of K_n_F_m_K_n_ peptides and its targeting mechanism were discussed. By synthesis, each amino acid of the K_n_F_m_K_n_ peptide was added sequentially to the peptide of interest, which allowed us to precisely modify the sequences to modulate their anticancer potency and investigate the structure–activity relationship of the AMPs’ primary sequence variations. This structure–activity relationship analysis was essential to further develop and to efficiently screen AMPs with fine-tuned selectivity for cancer cell membranes.

## 2. Materials and Methods

### 2.1. Reagents

The four peptides used in this study, namely, K_2_F_6_K_2_, K_3_F_6_K_3_, K_4_F_6_K_4_, and K_4_F_8_K_4_, were the same batch as those previously described. Thus, we had already confirmed their antibacterial effects both in vitro and in vivo [24]. Their basic properties are displayed in Table 1. The peptide was dissolved in sterile 1X phosphate-buffered saline (PBS, Corning, Corning, NY, USA). Paraformaldehyde (PFA) was purchased from USA Sigma Company (St. Louis, MO, USA), and prepared by ddH_2_O with a working concentration of 4% (*w/v*). Cell Counting Kit-8 (CCK-8) was purchased from Dojindo Company (Kumamoto, Japan). The live/dead assay kit (Calcein AM/PI) was purchased from KeyGen Biotech (Nanjing, China). The AnnexinV-FITC/PI cell apoptosis kit was purchased from Shanghai Yeason (Shanghai, China). The CellMask™ Deep Red plasma membrane stain kit was purchased from Molecular Probes, Life Technologies. The glass-bottom cell culture dish was purchased from NEST SCIENTIFIC INC. Matrigel was purchased from BD Biosciences and diluted by PBS with 1:50 (*v/v*).

### 2.2. Cell Culture

The human lung adenocarcinoma A549 and normal human lung fibroblast (HLF) cells were kindly provided by the National Collection of Authenticated Cell Cultures (Shanghai, China). A549 cells were cultured in the medium containing 88% Dulbecco’s Modified Eagle Medium with Ham’s F12 (DMEM/F12, Gibco, Waltham, MA, USA), 10% fetal bovine serum (FBS, Gibco), 1% Glutamax (Invitrogen, Waltham, MA, USA), and 1% penicillin/streptomycin (Gibco). The medium of HLF cells was the same as that of A549, except 88% DMEM (Gibco, 11965). All cells were cultured at 37 °C in an incubator (Thermo Fisher Scientific, Inc., Waltham, MA, USA) with 5% CO_2_. The media were changed every 2–3 days. When cells reached 80% confluence, subculturing was performed by 0.05% trypsin-EDTA (Gibco) at a ratio of 1:3. HLF cells of passages 2–6 (P2-6) were used for performing all subsequent experiments.

### 2.3. CCK-8 Analysis

For the cell viability assessment, cells were plated in a 96-well plate (about 5 × 10^3^ cells/well), with 5 replicates per group. Twenty-four hours after seeding, both A549 and HLF cells were treated with increasing concentrations of AMPs (0, 62.5, 125, 250, 500, and 1000 μg/mL) for 4, 24, and 48 h, respectively. Furthermore, all cells were incubated for 2.5 h under 37 °C with 10 μL CCK-8 solution. The absorbance at 450 nm represented a direct correlation with the cell number in this analysis and was measured using a standard microplate reader (Thermo-Scientific Varioskan Flash, Waltham, MA, USA). The inhibitory percentage of each peptide at various concentrations was calculated to determine the half maximum inhibitory concentration (IC_50_) value.

Cell viability (%) = [(OD_treat_ − OD_blank_)/(OD_control_ − OD_blank_)] × 100%.

The inhibition rate of cell growth (%) = [(OD_control_ − OD_treat_)/(OD_control_ − OD_blank_)] × 100%.

### 2.4. Live/Dead Staining of Cells

Live/dead staining was performed to analyze the effects of different K_4_F_6_K_4_ concentrations on cell viability. Briefly, 5 × 10^4^ cells were seeded into 24-well plates. The attached cells were then supplemented with peptide at 0, 62.5, 125, 250, 500, and 1000 μg/mL and incubated for 12 h, respectively. Furthermore, the cells were stained according to the manufacturer’s instructions. Finally, fluorescence microscopic images were taken from all the samples using an inverted phase-contrast fluorescence microscope (Nikon Eclipse Ti-S, Tokyo, Japan).

### 2.5. Colony Formation Assay

A total of 400 cells were seeded into 6-well plates, with 3 replicates per group. After the cells were attached, K_4_F_6_K_4_ was incubated, with increasing concentrations of peptide (0, 62.5, 125, 250, 500, and 1000 μg/mL) for 10 days. Then, the cells were fixed at 4% PFA for 15 min and stained with 1% crystal violet (solarbio, G1062) for 30 min at room temperature. The cell colonies (more than 50 cells) were counted, which indicated the ability of cell colony formation. The assay was conducted three independent times.

Colonies (%) = colony number of treatment group/colony number of control group × 100%.

### 2.6. Apoptosis Analysis

An AnnexinV-FITC/PI staining kit was used to test apoptosis. Accordingly, 5 × 10^5^ cells were seeded in a 6-well plate. The next day, K_4_F_6_K_4_ was incubated with increasing concentrations of peptide (0, 62.5, 125, 250, 500, and 1000 μg/mL). After 12 h incubation, the cells were collected and washed twice with PBS. In accordance with the kit specification, the cells were stained by Annexin V and propidium iodide (PI), then evaluated by fluorescence-activated cell sorting analysis (BD Facsaria III; BD Biosciences, San Jose, CA, USA).

### 2.7. Membrane Staining and Confocal Imaging

Next, 2 × 10^5^ cells were seeded in the glass-bottom cell culture dish coated with matrigel overnight. After incubating for 0, 4, and 8 h with the addition of 125 μg/mL K_4_F_6_K_4_, cells were labeled with 1 µg/mL of the CellMask™ Deep Red plasma membrane dye for 10 min at 37 °C. Then, after being washed three times with DMEM/F12, the cells were fixed at 4% PFA for 10 min at room temperature, followed by washing three times with PBS. Cell nuclei were stained with 4′,6-diamidino-2-phenylindole (DAPI, Invitrogen) for 30 min and rinsed three times with PBS. Finally, images were captured by Leica TCS SP8 confocal fluorescence microscope.

### 2.8. Scanning Electron Microscopy (SEM)

Next, 2 × 10^4^ A549 or HLF cells were seeded into 24-well plates, in which each well contained one coverslip. The attached cells were then supplemented with 125 μg/mL K_4_F_6_K_4_ and incubated for 12 h. For SEM analysis, the cells were fixed at 4% PFA for 20 min at room temperature and washed with PBS. Then, dehydration was performed in 70%, 80%, and 95% ethanol (Sigma) for 20 min with each concentration. After being dried by carbon dioxide, all coverslips were observed under a scanning electron microscope (Zeiss, Berlin, Germany).

### 2.9. Xenograft Tumor Model

A total of 10 BALB/c nude mice (females, 6 weeks old, body weight 18–20 g) were purchased from the experimental animal center at Tongji University (Shanghai, China). The protocols for animal studies were approved by the Animal Experimental Ethical Inspection department of the Laboratory Animal Centre, Tongji University (2021tjdx087). Then, 3 × 10^6^ A549 cells were implanted into the right flank of mice via subcutaneous injection. Tumor sizes were calculated using a Vernier caliper, as follows: tumor volume (mm^3^) = (L × W^2^)/2, where L = long axis and W = short axis. Once the tumor size was 4 mm in diameter (this took ten days in this study), either K_4_F_6_K_4_ (100 μL, 10 mg/kg) or PBS (100 μL) were injected intraperitoneally, every other day. On the 29th day of the experiment, euthanized animals were then subjected to cervical dislocation to ensure euthanasia, and excised xenograft tumors were weighed. Liver tissues were fixed by 4% PFA and histologically reviewed by hematoxylin and eosin (HE) staining. All mice were weighed every other day.

### 2.10. Statistical Analysis

Statistical analyses were performed using SPSS 25.0 (IBM Corporation, Armonk, NY, USA) and GraphPad Prism 9.0 (GraphPad software, San Diego, CA, USA). All data were analyzed by Student’s *t*-test and one-way analysis of variance (ANOVA). All data are expressed as mean ± standard deviation and obtained from at least three independent experiments. *p* < 0.05 was considered to indicate a statistically significant difference and *p* < 0.05, <0.01, and <0.001 are indicated as *, **, and *** in the figures, respectively.

## 3. Results

### 3.1. Preferential Cytotoxicity of K_4_F_6_K_4_ against Tumor Cells by CCK-8 and Live/Dead Assays

**Figure 1:**
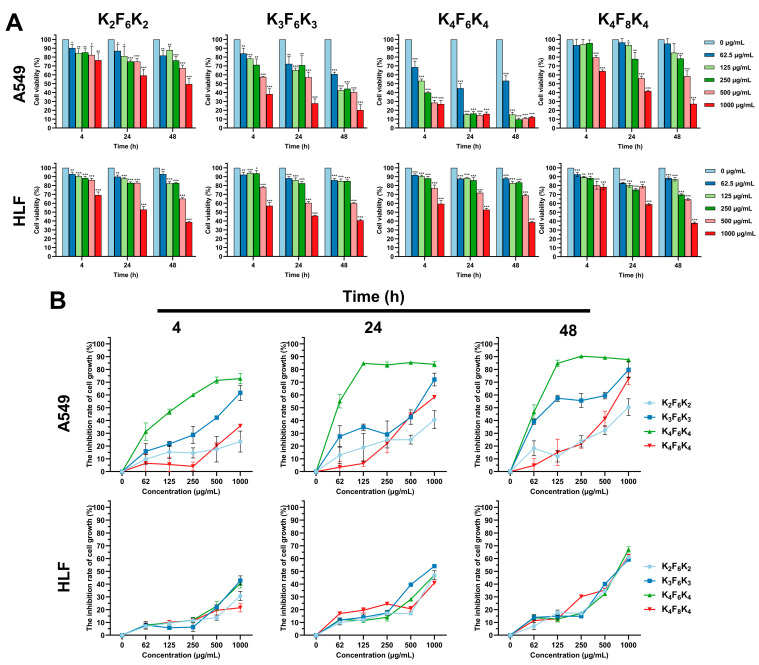
Preferential cytotoxicity of the triblock amphiphilic short AMPs (K_n_F_m_K_n_: K_2_F_6_K_2_, K_3_F_6_K_3_, K_4_F_6_K_4_, and K_4_F_8_K_4_) with different concentrations (0, 62.5, 125, 250, 500 and 1000 μg/mL) for 4, 24 and 48 h against A549 cells by CCK-8, with HLF as the negative control. (**A**) Cell viability rate of A549 and HLF cells after being treated with K_n_F_m_K_n_; (**B**) The inhibition rate of A549 and HLF cell growth after being treated with K_n_F_m_K_n_. Data are expressed as the mean ± standard deviation. * *p* < 0.05, ** *p* < 0.01 and *** *p* < 0.001 vs. 0 μg/mL group.

To explore the antitumor activity and selectivity profile of the triblock amphiphilic short AMP analogues (K_n_F_m_K_n_: K_2_F_6_K_2_, K_3_F_6_K_3_, K_4_F_6_K_4_, and K_4_F_8_K_4_), a cell viability assay was conducted in the human lung adenocarcinoma cell line A549 by CCK-8, with HLF cells as the negative control. The time and dose effects of these four peptides were studied systematically. Notably, from the information given in Figure 1A, when the four peptides were at the same concentration and the same time point, the percentage of cell viability corresponding to A549 with K_4_F_6_K_4_ treatment was the smallest. Additionally, the inhibition rate of cell growth in the K_4_F_6_K_4_ treatment group exhibited the most dramatic increase at the same time point in Figure 1B. In addition, the cytotoxicity concentrations of four peptides required to kill A549 cells by 50% (IC_50_) were calculated in Table 2 from Figure 1A. As expected, K_4_F_6_K_4_ exhibited the least IC_50_ with the value of 62.64 ± 9.55 μg/mL at 48 h. It was indicated that A549 cells were much more sensitive to K_4_F_6_K_4_ than the other three peptides. In contrast, it was less toxic to noncancer cells (HLF), with an IC_50_ value of 808.82 ± 125.7 μg/mL at 48 h. Additionally, the toxicity of K_4_F_6_K_4_ towards cancer cells was generally about 13 times higher than that towards noncancer cells. Of note, beyond 250 μg/mL, although the cell growth inhibition rate was still at the highest level in the A549 group, it increased obviously in the HLF group. That is to say, K_4_F_6_K_4_ preferentially suppressed cancer cell lines within the dosage range <250 μg/mL, while its cytotoxic effect on normal cells was low (<14%) within this range.

It has been reported that a net positive charge is an important and common feature impacting the effects of AMPs [25]. Based on Table 2, evidently, increasing the number of the positively charged lysine residues of K_n_F_6_K_n_ could promote its antitumor activity, since the IC_50_ values of the peptides clearly declined: K_4_F_6_K_4_ < K_3_F_6_K_3_ < K_2_F_6_K_2_. In other words, in the same setting, for K_2_F_6_K_2_, K_3_F_6_K_3_, and K_4_F_6_K_4_, the difference in the inhibitory effect depended on the difference in the cationic charging effects. Thus, it was deduced that increasing the cationic charging effects of K_n_F_6_K_n_ could promote antitumor activity and selectivity, and the antitumor activity of K_4_F_6_K_4_ was supported by its cationic charging effects. Despite sharing the same number of cationic charges, K_4_F_8_K_4_ exhibited much weaker cytotoxicity than that of K_4_F_6_K_4_ under the same working conditions. Therefore, the hydrophobic part was another driving force contributing to the cytotoxic effects of K_4_F_6_K_4_, while more hydrophobic groups were not always better.

**Figure 2:**
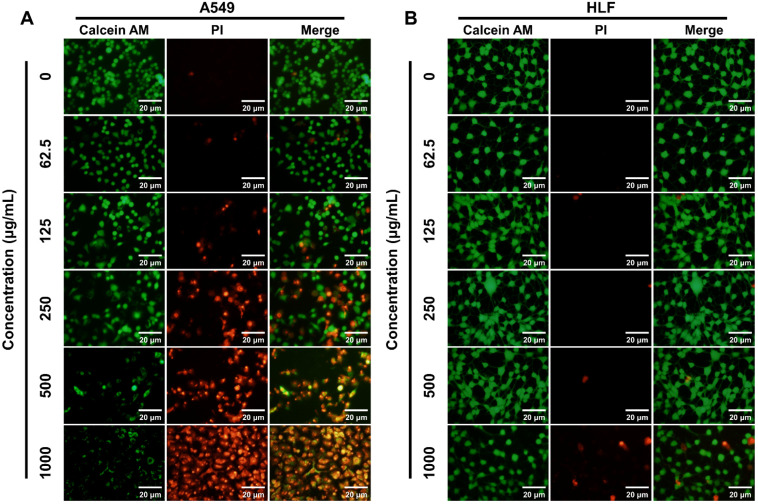
(**A**) A549 and (**B**) HLF cells with different concentrations of K_4_F_6_K_4_ for 12 h and dyed by calcein AM and PI to distinguish live cells from dead cells. The green fluorescence represents living cells and the red fluorescence represents dead cells. The scale bar represents 20 μm.

As previously mentioned, K_4_F_6_K_4_ could efficaciously kill A549 cells but not normal cells; therefore, it was selected for further characterization in the following assay. To visually evaluate the tumoricidal ability of K_4_F_6_K_4_, A549 and HLF cells with different concentrations of K_4_F_6_K_4_ were dyed with calcein AM and PI to distinguish live cells from dead cells. In Figure 2A, it can be observed that the higher the concentration of K_4_F_6_K_4_, the more cancer cells died. The use of 250 μg/mL K_4_F_6_K_4_ led to a mortality rate of about 35% for A549 cells. Moreover, the addition of 500–1000 µg/mL K_4_F_6_K_4_ resulted in almost entirely dead A549 cells. Regarding the controls, it can be seen from Figure 2B that there were no detectable red fluorescence signals in any of the ≤250 μg/mL K_4_F_6_K_4_-treated groups, which indicates that drug concentrations in this range did not cause noticeable HLF cell death. Even with the higher drug concentrations (500–1000 µg/mL) the sporadic red fluorescence signals suggested little non-cancerous cell loss. This phenomenon further demonstrates that the cytotoxicity of K_4_F_6_K_4_ against A549 was preferential.

### 3.2. K_4_F_6_K_4_ Inhibited the Colony Formation of Lung Cancer Cells

**Figure 3:**
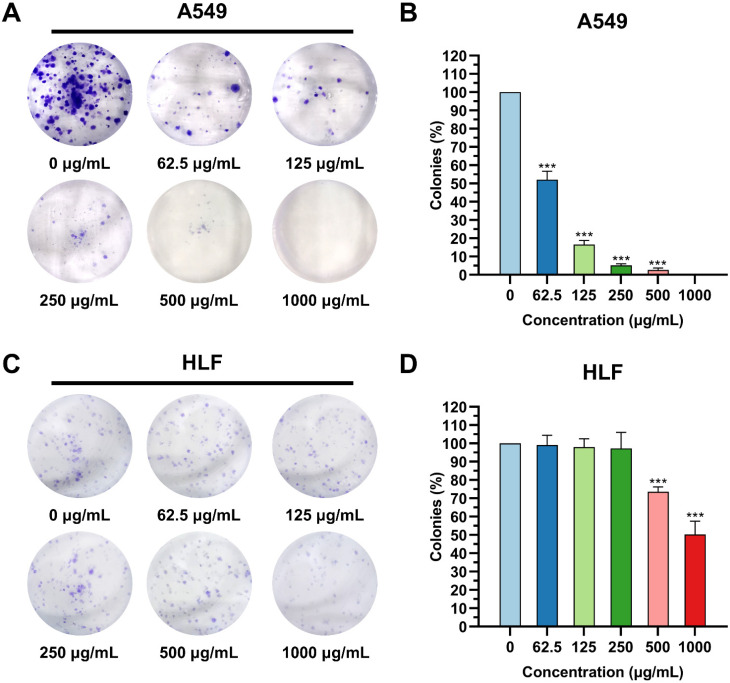
The inhibitory effect of K_4_F_6_K_4_ on the proliferation capacity of A549 was detected by the colony formation assay, with HLF as the negative control. (**A**) Images of A549 colonies with different concentrations of K_4_F_6_K_4_; (**B**) statistical analysis of the colony numbers in the A549 group; (**C**) images of HLF colonies with different concentrations of K_4_F_6_K_4_; (**D**) statistical analysis of the colony numbers in the HLF group. Data are expressed as the mean ± standard deviation. *** *p* < 0.001 vs. 0 μg/mL group.

The inhibitory effect of K_4_F_6_K_4_ on the proliferation capacity of A549 was detected by the colony formation assay, with HLF as the negative control. Clearly, in Figure 3A,B, the anti-proliferation ability of K_4_F_6_K_4_ on A549 was inversely proportional to the colony formation rate. After being treated with 62.5 μg/mL K_4_F_6_K_4_, the colony formation rate decreased sharply to 52.03 ± 4.67%, and no obvious colony formation took place with >125 μg/mL K_4_F_6_K_4_ used for treatment, owing to the very low colony formation rate below 10%. In comparison, in Figure 3C,D, the colony formation rate for normal cell HLF was not disturbed within the effective concentration (<250 μg/mL). This means that K_4_F_6_K_4_ selectively inhibited the cellular growth of cancerous cell line A549 without obviously decreasing the cellular proliferation activity of the non-cancerous cell line HLF. This result was coincident with that of CCK-8.

### 3.3. K_4_F_6_K_4_ Induced Lung Cancer Cell Apoptosis

**Figure 4:**
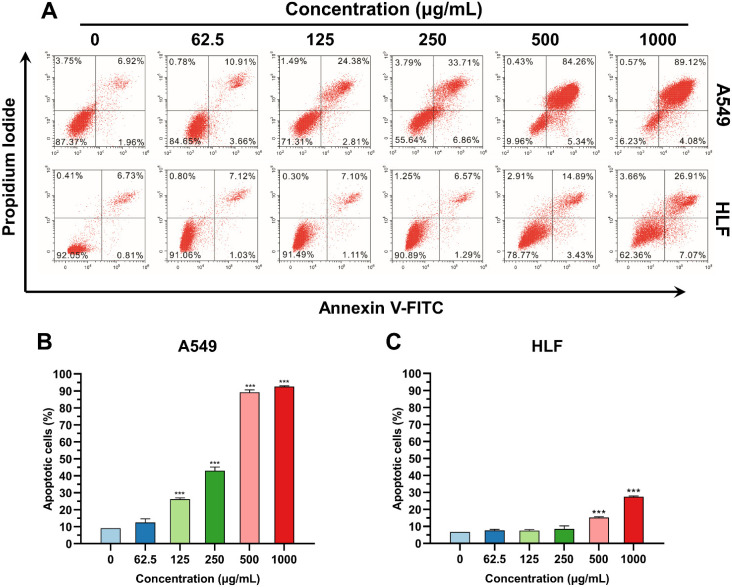
AnnexinV-FITC/PI double staining followed by flow cytometry was undertaken to assess the integrity of the cell membrane. (**A**) Representative flow cytometry plots suggest the apoptosis of A549 and HLF cells treated with different concentrations of K_4_F_6_K_4_ for 12 h. The upper right quadrant (Annexin V^+^/PI^+^) represents the late apoptotic stage cells and the lower right quadrant (Annexin V^+^/PI^-^) represents the early apoptotic stage cells. The total proportion of apoptotic cells (early and late) in the (**B**) A549 group and (**C**) HLF group. Data are expressed as the mean ± standard deviation. *** *p* < 0.001 vs. 0 μg/mL group.

Normally, phosphatidylserine (PS) is restricted to the inner leaflet of the plasma membrane; however, lipid asymmetry is lost during apoptosis and PS becomes exposed on the outer leaflet of the plasma membrane [26,27,28]. K_4_F_6_K_4_ preferentially kills cancer cells, although the mechanism remains unclear. To explore the preliminary antitumor mechanism, an AnnexinV-FITC/PI double staining followed by flow cytometry was undertaken to assess the integrity of the cell membrane and the externalization of PS. Flow cytometry analysis showed that the exposure of K_4_F_6_K_4_ did not induce an obvious increase in the levels of A549 cell necrosis; however, it conducted significant apoptosis (Figure 4A). The total proportion of apoptotic cells (early and late) increased in a dose-dependent manner. Within the dosage range <250 μg/mL, the number of A549 apoptotic cells (early and late) increased from 9.59 ± 1.00% to 43.03 ± 2.13% (Figure 4B and Table 3); on the other hand, a negligible apoptosis increase in HLF was induced from 7.87 ± 0.99% to 9.85 ± 1.75% (Figure 4C and Table 3). In the case of exposure to 500–1000 μg/mL, the percentages of apoptotic A549 were about 3–5-fold higher than that of HLF (89.30 ± 1.41% vs. 18.82 ± 0.51% and 92.66 ± 0.46% vs. 34.28 ± 0.27%). Cell apoptosis was the predominant cell death type. In relation to the above-mentioned determinations, the study proved that, statistically, K_4_F_6_K_4_ effectively induced the cellular apoptosis of A549 and contributed to suppressing tumor cells, compared to the control cells.

### 3.4. K_4_F_6_K_4_ Noticeably Disrupted A549 Cell Membrane Integrity and Induced Morphological Modifications

**Figure 5:**
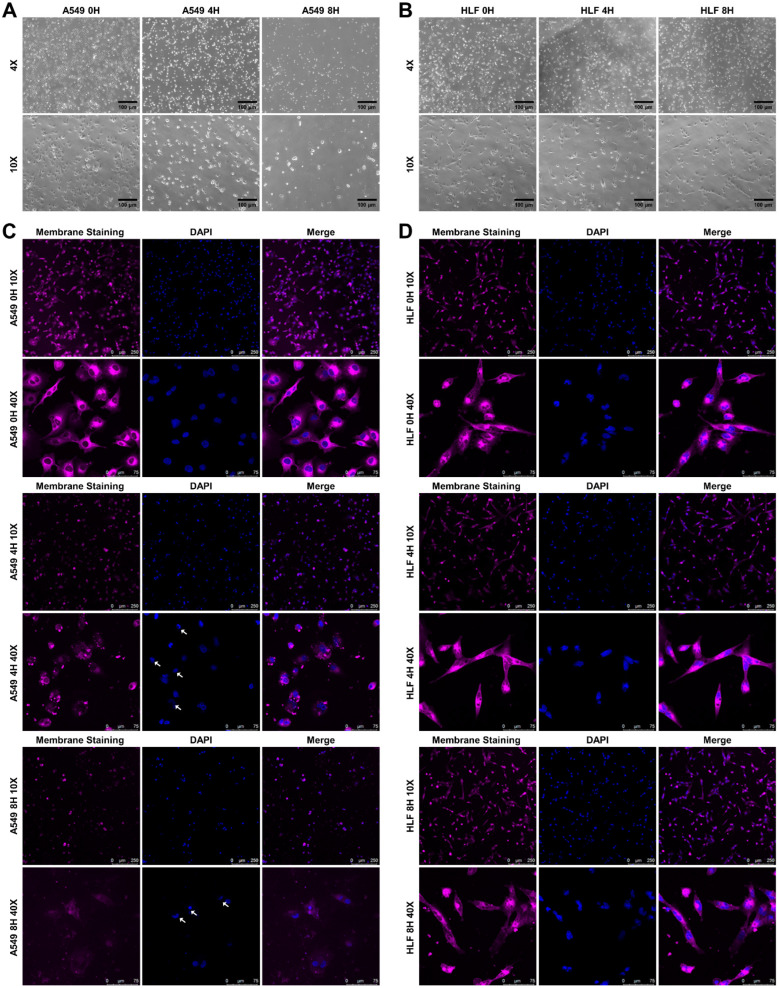
Morphological changes of (**A**) A549 and (**B**) HLF cells treated with K_4_F_6_K_4_ for 0, 4, and 8 h were observed by optical microscopy. Additionally, the cell plasma membrane and nuclei of (**C**) A549 and (**D**) HLF cells treated with K_4_F_6_K_4_ for 0, 4, and 8 h were stained with CellMask™ Deep Red plasma membrane dye and DAPI, respectively. The white arrows show the cells with typical apoptotic features after DAPI staining.

To continue our investigation, we investigated the status of the plasma membrane with three instruments. To begin with, the cell morphology changes of the cells exposed to K_4_F_6_K_4_ were observed using an optical microscope. As seen from Figure 5A, K_4_F_6_K_4_ treatment not only evidently reduced the density of A549 cells in a time-dependent manner, but also induced noticeable morphological modifications. After being treated with K_4_F_6_K_4_ for 4 h, many cells displayed signs of apoptosis, i.e., shriveled membranes and round cell bodies, when compared to the untreated control cells. When the cells were incubated for 8 h with the peptide of interest, a large amount of vacuolation occurred and cell debris appeared in the cell supernatant, indicating the occurrence of necrosis. Therefore, we hypothesized that the K_4_F_6_K_4_ inhibition of tumor cell growth may be involved in both apoptosis and necrosis. In contrast, K_4_F_6_K_4_ treatment did not reduce the density of HLF cells or change the non-cancerous cells’ morphologies (Figure 5B).

Next, the integrity of the cellular plasma membrane was monitored by confocal microscopy with CellMask™ Deep Red staining. In the absence of peptides at 0 h, as revealed by the CellMask™ Deep Red imaging, A549 cells were small, round, or polygonal (Figure 5C), and the HLF cells had a uniform spindle pattern (Figure 5D). The overall staining status of both A549 and HLF cells exhibited intact membrane structure. Significant morphological alterations were observed as early as 4 h after K_4_F_6_K_4_ treatment for A549 cells (Figure 5C). The membrane boundary of the majority of A549 cells became dim and discrete, indicating that the membrane was broken. In detail, as shown by the white arrows, some of the A549 cells displayed condensed nuclei from DAPI staining, a characteristic morphology of apoptosis. Additionally, the cytotoxicity increased dramatically as time went on. Upon K_4_F_6_K_4_ exposure for 8 h, a remarkable decrease in the red fluorescence positive membrane of the A549 cells was found. Almost all the cells were broken into pieces. It should be mentioned that dead cells might detach from the plate; in this case, the decrease in the red fluorescence could also indicate cell loss. Comparatively, no detectable morphological alterations were caused by K_4_F_6_K_4_ against HLF cells under the same condition.

**Figure 6:**
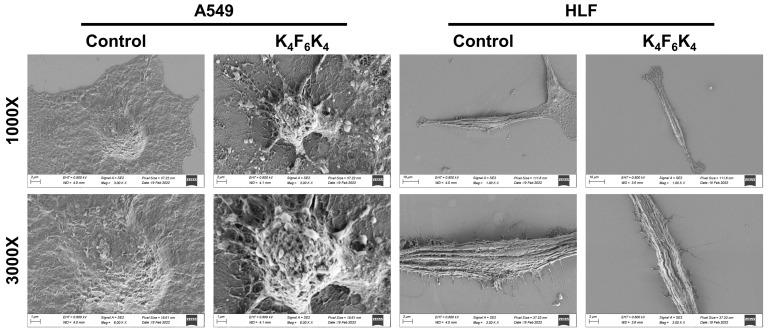
SEM images of the A549 and HLF cells treated with K_4_F_6_K_4_ for 12 h. Magnifications were 1000× and 3000×.

The morphological observation using SEM once again revealed that the incubation of A549 with K_4_F_6_K_4_ induced dramatic morphological changes. As seen in Figure 6, untreated A549 cells showed an intact membrane with rich microvilli, curl plexiform, regular arrays, and filament distribution. However, K_4_F_6_K_4_ treatment resulted in cell body shrinkage, the curling and surface distortion of the microvilli, gradually increasing ball structure, missing parts of the microvilli cell surface, and an invaginated membrane. These may, in turn, result in irreversible cytolysis and finally the death of the target cells. However, the addition of K_4_F_6_K_4_ did not produce any harmful effects on the non-cancerous cells HLF.

To summarize, our data suggest that K_4_F_6_K_4_ might interact with the lipids or proteins on the plasma membrane, and the resulting membrane disruption is thought to be the primary mechanism of its antitumor action.

### 3.5. K_4_F_6_K_4_ Significantly Inhibited Tumor Growth in Xenografted Mice without Measurable Side Effects

**Figure 7:**
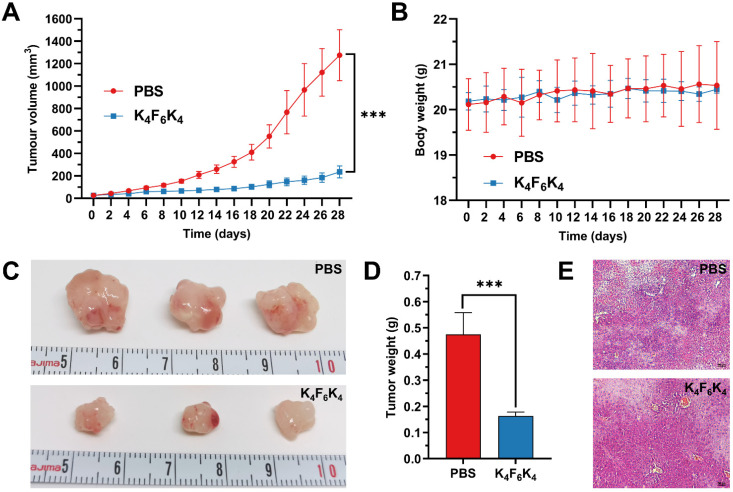
The therapeutic effects of K_4_F_6_K_4_ on tumor growth in nude BALB/c mice. (**A**) The mean tumor volume and (**B**) body weight of mice during the treatment process. (**C**) Photos of the excised tumors after the treatment of PBS and K_4_F_6_K_4_. (**D**) Statistical analysis of the excised tumor weights after the experiment. (**E**) HE staining of the liver tissue with the treatment of PBS and K_4_F_6_K_4_ after the experiment. Data are expressed as the mean ± standard deviation. *** *p* < 0.001 vs. the PBS group.

To investigate the *in vivo* therapeutic efficacy of K_4_F_6_K_4_, we performed experiments using xenografted A549 tumor cells on nude mice. Ten days after the subcutaneous injection of the A549 cells, the tumor volume was around 26 mm^3^ in each group before the treatment started. Then, the mice were randomized into two groups (*n* = 5 mice/group) for PBS and K_4_F_6_K_4_ treatment. These mice were administered drugs by peritoneal injection every other day. In the control group, PBS was injected. In the constructed xenograft tumor model, K_4_F_6_K_4_ treatment affected tumor growth significantly (Figure 7A,C). The tumor grew considerably quickly and reached 1275.38 ± 226.6 mm^3^ 28 days after receiving treatment in the PBS group. Although the tumor still grew slowly during K_4_F_6_K_4_ treatment and reached 235.29 ± 52.93 mm^3^ after the same treatment period, the tumor growth was substantially reduced. The tumor volume was reduced by 81.6%. In addition, the mean tumor weight of each group was measured after the treatment was terminated (Figure 7D). The heaviest tumor (0.47 ± 0.08 g) was found in the control group, and it was three times higher than that (0.16 ± 0.02 g) of the K_4_F_6_K_4_ treatment group. The results indicated that the weights of the excised tumors were significantly reduced by K_4_F_6_K_4_. Moreover, the hematoxylin and eosin (HE) staining of the liver tissue and the weight of the mice were recorded after the treatment with K_4_F_6_K_4_ (Figure 7B,E). No harmful effects were found in relation to the liver or body weight of the K_4_F_6_K_4_-treated mice.

## 4. Discussion

As is already known, lung cancer is one of the most frequently occurring diseases in the world. Despite developing various therapeutic interventions, lung cancer remains a major cause of morbidity and mortality, imposing a heavy social and economic burden on individuals, families, communities, and countries [29]. The development of lung cancer drugs is still in pressing demand. The emerging threat of limited selectivity in tumor targeting and the emergency of resistance in tumor cells have motivated researchers to develop new classes of anticancer agents that can efficiently eliminate cancerous cells with no detrimental effects on adjacent healthy host cells [30].

In recent decades, there has been a surge of research focused on AMPs and their potential as therapeutics to combat pathogenic microorganisms. This has dramatically improved our understanding of these peptides’ specific functions. Some studies have proposed the anticancer capacity and tumoricidal effects of AMPs beyond their antipathogenic activity [31,32]. Importantly, they have the ability to bypass the mechanisms of excessive resistance and have no obvious toxicity to normal cells [33]. Inspired by this, the overall objectives of the present study were to assess the potent anticancer capacities of our newly designed K_n_F_m_K_n_ analogues (K_2_F_6_K_2_, K_3_F_6_K_3_, K_4_F_6_K_4_, and K_4_F_8_K_4_) against the lung cancer cell A549. According to the results from the CCK-8 assay, it can be concluded that the anticancer activity of K_4_F_6_K_4_ was markedly more potent than that of the other three peptides. Within the effective dose range of 125–250 μg/mL, the inhibition rate of K_4_F_6_K_4_ on A549 cell growth stayed at the highest level. It is worth noting that the IC_50_ value of K_4_F_6_K_4_ against noncancer cells was about 13 times greater than that against cancer cells. Moreover, K_4_F_6_K_4_ demonstrated no significant adverse effects on non-cancerous cells and selectively eliminated cancer cells within the effective dose range. This was also confirmed by the live/dead cell staining and colony formation assay. Importantly, the tumor size in vivo was significantly reduced by administering 10 mg/kg K_4_F_6_K_4_ every other day to BALB/c nude mice subcutaneously inoculated with A549 tumor cells. No harmful effects were caused by K_4_F_6_K_4_ to the liver or body weight of the treated mice. Consequently, the triblock amphiphilic short peptide K_4_F_6_K_4_, which has been identified to possess dual antimicrobial and anticancer activities without obvious side effects both in vitro and in vivo, is a good candidate for the development of anticancer agents.

**Figure 8:**
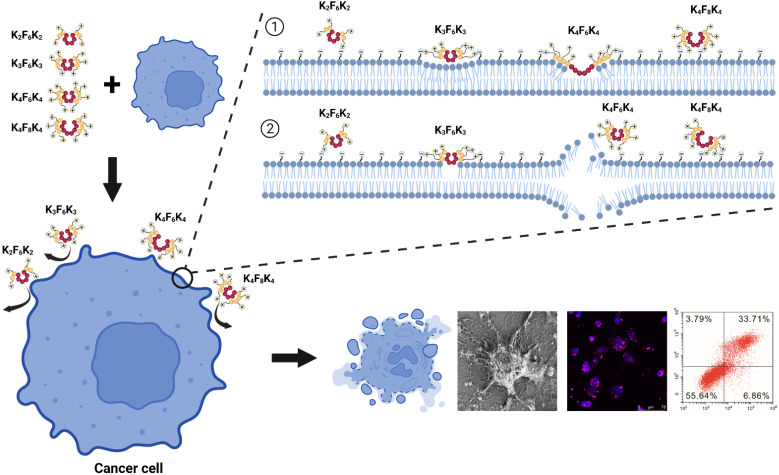
Schematic diagram of membrane disruption induced by K_n_F_m_K_n_ analogues (K_2_F_6_K_2_, K_3_F_6_K_3_, K_4_F_6_K_4_, and K_4_F_8_K_4_) against lung cancer cells A549. ① Firstly, the cationic K_n_F_m_K_n_ molecules were initially associated with the anionic cellular membranes via electrostatic interactions. During this process, increasing the cationic charging effects of K_n_F_6_K_n_ was found to promote antitumor activity. In addition, the tumor cells had elevated negative charges on the surface distinguished from noncancer cells; thus, K_4_F_6_K_4_ could selectively kill A549 cells, but not HLF cells. ② Secondly, the hydrophobicity of phenylalanine residues in the middle promoted the interaction between the peptides and the fatty acyl chains to form pores, to align parallel to the surface on the cell membrane, and to disrupt the cell membrane. This may, in turn, result in irreversible cytolysis and finally the death of the target cells. The cell apoptosis assay by flow cytometry, the morphology observations using the optical microscope, the confocal microscopy with the CellMask™ Deep Red staining, and the scanning electron microscope identified this point. Of note, there was an optimal hydrophobicity needed for bioactivity; sequences with hydrophobicities below or very much above this threshold made the peptides inactive. Therefore, K_4_F_8_K_4_ exhibited a much weaker cytotoxicity than that of K_4_F_6_K_4_ under the same working conditions. Phenylalanine (F) is represented by the red spheres and lysine (K) is represented by the yellow spheres. The number of spheres indicates the number of corresponding amino acids.

Although the mechanisms of AMPs exhibiting anticancer activity have not yet been studied thoroughly, it is certain that the activity of a peptide is directly related to its primary sequence. By synthesizing, we precisely modified the sequences and altered certain peptide characteristics of K_n_F_m_K_n_ analogues to modulate their anticancer potencies and investigate the primary structure–activity relationships. Here, the K_n_F_m_K_n_ analogues we designed were triblock amphiphilic short peptides with net cationic charges, in which hydrophilic and hydrophobic amino acids were segregated spatially by using positively charged lysine residues at both ends and hydrophobic phenylalanine residues in the middle. These characteristic features, which are in common with natural AMPs, are associated with their ability to kill lung cancer cells, as illustrated in Figure 8. Firstly, the cationic K_n_F_m_K_n_ molecules were initially associated with the anionic cellular membranes via their electrostatic interactions. Based on the outcomes of the CCK-8 assay, the IC_50_ values of the peptides clearly declined with the increasing number of cationic charges: K_4_F_6_K_4_ < K_3_F_6_K_3_ < K_2_F_6_K_2_. Evidently, increasing the cationic charging effects of K_n_F_6_K_n_ could promote its antitumor activity. In addition, the tumor cells had elevated negative charges on the surface distinguished from noncancer cells [34,35]; thus, K_4_F_6_K_4_ can selectively kill A549 cells, but not HLF cells. Therefore, it is deduced that cationicity might be the initiating factor in the interaction of K_4_F_6_K_4_ with A549 cell membranes. Secondly, the hydrophobicity of the phenylalanine residues in the middle promoted the interaction between the peptides and the fatty acyl chains to form pores, to align parallel to the surface on the cell membrane, and to disrupt the cell membrane. This may, in turn, result in irreversible cytolysis and finally the death of the target cells. Our confocal imaging results demonstrated that the A549 cell membrane stained by CellMask™ Deep Red dye was damaged as early as 4 h after treatment with K_4_F_6_K_4_; however, in the HLF case, the cell membrane was intact. This observation was confirmed by SEM, which showed that K_4_F_6_K_4_ treatment resulted in cell body shrinkage, the curling and distortion of the microvilli surface, a gradual increase in ball structure, missing parts of the microvilli cell surface, and membrane invagination. The addition of K_4_F_6_K_4_, on the other hand, did not produce any harmful effect on the non-cancerous cells (HLF). Consistent with these findings, the morphological observations using the optical microscope demonstrated that K_4_F_6_K_4_ treatment not only evidently reduced the density of the A549 cells, but also induced noticeable apoptosis with shriveled membranes and necrosis. For comparison, HLF cells were kept intact. The cell apoptosis assay utilizing AnnexinV-FITC/PI double staining offered another piece of evidence for this anticancer mechanism. As indicated by the flow cytometry results, the A549 cell membranes were disrupted after treatment with K_4_F_6_K_4_: the number of late-stage apoptotic cells accounted for the majority of the whole counting cells. The HLF cell membranes were intact after treatment with K_4_F_6_K_4_: the number of late-stage apoptotic cells was not obviously changed. Of note, hydrophobicity was another key feature for all AMPs, defined as the percent of hydrophobic residue in the peptide sequence [36,37]. An optimal hydrophobicity was needed for bioactivity, and sequences with hydrophobicities below or very much above this threshold made the peptides inactive [37,38,39]. Therefore, K_4_F_8_K_4_ exhibited a much weaker cytotoxicity than that of K_4_F_6_K_4_ under the same working conditions.

To obtain AMPs with low toxicity, strong activity, and diverse functions, it is necessary to further study the structure–function relationship of AMPs. Fortunately, the triblock amphiphilic short K_n_F_m_K_n_ peptides we designed were successfully developed with dual functions; thus, we can move forward to explore the scientific problem of “the connections between the primary sequence of K_n_F_m_K_n_ peptides and its targeting mechanism”. The tumoricidal activity shared the membrane disruption mechanism with the antimicrobial process, in which the main driving forces for membrane binding were electrostatic attraction and hydrophobic interaction [35,40,41]. Based on our comprehensive study, our results indicate that the action mechanism for K_n_F_m_K_n_ interactions with A549 cancer cells can be considerably affected by slight differences from that with microbes. Specifically, among the four K_n_F_m_K_n_ analogues, K_4_F_6_K_4_ exhibited the best effects in the tumoricidal process. This matched the charging effects theory: the more cationic charge, the stronger the affinity with the anionic biomembrane surface. Moreover, K_3_F_6_K_3_, but not K_4_F_6_K_4_, showed a higher efficiency in the antimicrobial process. This implies that a cationic charge does not always enhance activity; when beyond the optimum level of charge, increased charge reduces AMP activity [41,42]. Moreover, the amount of positive charge required for K_n_F_m_K_n_ to exert its optimal antibacterial effect was less than that required for it to exert its optimal antitumor effect. On the other hand, in terms of the antimicrobial activity of K_3_F_6_K_3_, the proportion of the hydrophobic phenylalanine residue was 50%, which was equal to the typical proportion value of most AMPs [37]. The proportion of the hydrophobic phenylalanine residue of K_4_F_6_K_4_, on the other hand, was about 43%. Therefore, the optimal hydrophobicity in the tumoricidal activity was less than that needed for the antimicrobial activity.

## 5. Conclusions

In conclusion, we identified that the triblock amphiphilic short K_4_F_6_K_4_ peptides we designed were successfully developed with dual functions via the membrane disrupted mechanism, and K_4_F_6_K_4_ has the potential to become an anticancer candidate. The tumoricidal activity of these K_n_F_m_K_n_ analogues shared the same mechanisms as their antimicrobial processes, and thus the main driving forces for membrane binding were electrostatic attraction and hydrophobic interaction. The action mechanism for K_n_F_m_K_n_ interactions with A549 cancer cells could be considerably affected by slight differences in microbes. Namely, the amount of positive charge required for K_n_F_m_K_n_ to exert its optimal tumoricidal effect was more than that needed for the antimicrobial activity, while the optimal hydrophobicity was less. Our findings suggest that further analysis of the structure–activity relationships of AMP primary sequence variations is necessary. Hopefully, this work can provide guiding principles in designing peptide-based therapeutics for lung cancer.

## Figures and Tables

**Table 1:** The basic properties of K_n_F_m_K_n_.

AMPs	Sequence	Molecular Weights	Proportion of the Hydrophobicity
K_2_F_6_K_2_	Lys-Lys-Phe-Phe-Phe-Phe-Phe-Phe-Lys-Lys	1431.9	60%
K_3_F_6_K_3_	Lys-Lys-Lys-Phe-Phe-Phe-Phe-Phe-Phe-Lys-Lys-Lys	1688.26	50%
K_4_F_6_K_4_	Lys-Lys-Lys-Lys-Phe-Phe-Phe-Phe-Phe-Phe-Lys-Lys-Lys-Lys	1944.66	43%
K_4_F_8_K_4_	Lys-Lys-Lys-Lys-Phe-Phe-Phe-Phe-Phe-Phe-Phe-Phe-Lys-Lys-Lys-Lys	2239.04	50%

**Table 2:** Cytotoxicity (IC_50_, μg/mL ± SD) of K_n_F_m_K_n_ against A549 and HLF cells at 48 h.

AMPs	A549	HLF
K_2_F_6_K_2_	1146.23 ± 346.83	758.66 ± 82.02
K_3_F_6_K_3_	123.10 ± 35.19	758.77 ± 105.3
K_4_F_6_K_4_	62.64 ± 9.55	808.82 ± 125.7
K_4_F_8_K_4_	571.87 ± 66.23	704.19 ± 78.34

**Table 3:** The percentage (mean ± S.D.) of apoptotic cells treated with K_4_F_8_K_4_.

Concentration (μg/mL)	A549	HLF
0	9.59 ± 1.00%	7.87 ± 0.99%
62	12.51 ± 2.23%	8.85 ± 0.74%
125	26.19 ± 0.89%	8.67 ± 0.92%
250	43.03 ± 2.13%	9.85 ± 1.75%
500	89.30 ± 1.41%	18.82 ± 0.51%
1000	92.66 ± 0.46%	34.28 ± 0.27%

## Data Availability

All data of this study are contained within the article.
